# Aspergillus encephalitis with microabscesses in an immunocompetent patient

**DOI:** 10.1590/0037-8682-0391-2023

**Published:** 2023-09-22

**Authors:** Recep Tekin, Salih Hattapoğlu, Rojbin Ceylan Tekin

**Affiliations:** 1 Dicle University, Faculty of Medicine, Department of Infectious Diseases and Clinical Microbiology, Diyarbakir, Turkey.; 2 Dicle University, Faculty of Medicine, Department of Radiology, Diyarbakir, Turkey.; 3 Mardin State Hospital, Department of Radiology, Mardin, Turkey.

**FIGURE 1:**
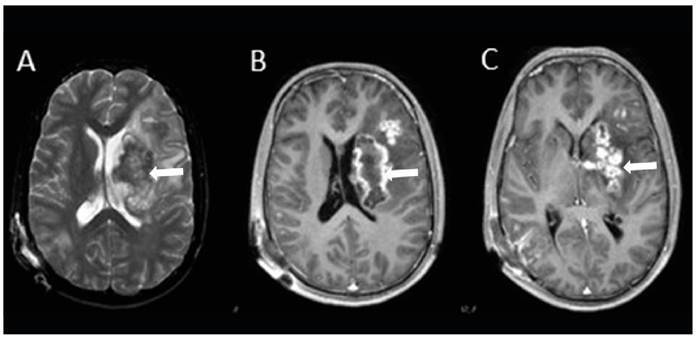
**A.** Brain MRI showingT2-weighted images large hypointense lesion causing a minimal mass effect, accompanied by significant peripheral edema. **B-C.** Solid components are hyperintense after contrast enhancement (arrows).

**FIGURE 2:**
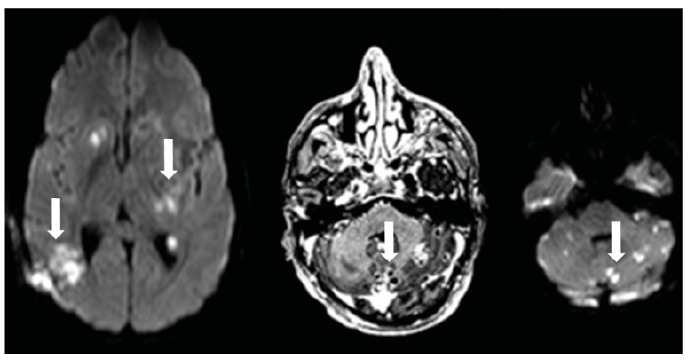
Diffusion-weighted imaging revealed several diffusely localized lesions with restricted diffusion in both hemispheres and the cerebellum (arrows).

**FIGURE 3:**
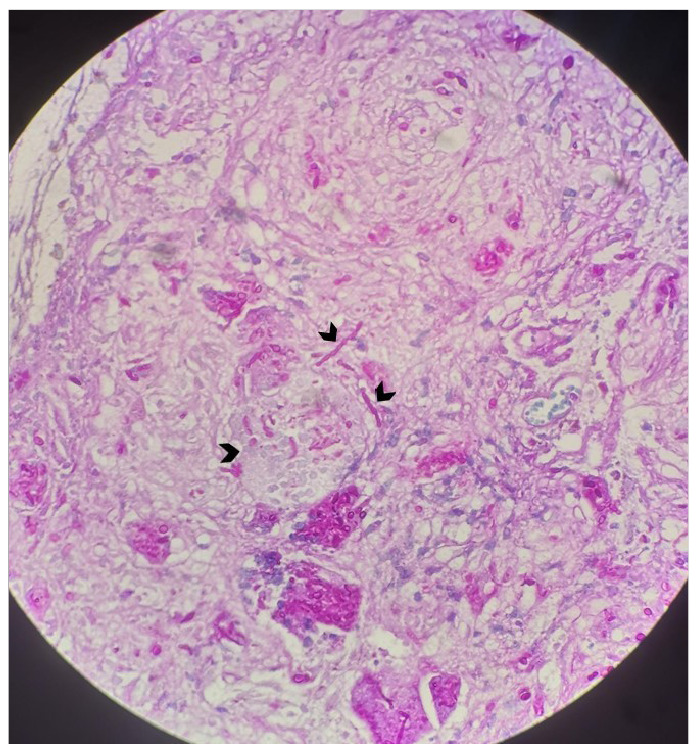
Pathological examination confirming the diagnosis of Aspergillus infection (arrowheads).

A 28-year-old man presented to the emergency department with a 5-week history of severe headache, dizziness, and weakness in the right arm. Neurological examination revealed 3/5 strength in the right arm. Brain magnetic resonance imaging (MRI) of the brain showed a large hypointense lesion with thick peripheral contrast enhancement in the left periventricular area. The solid components appeared hyperintense after contrast enhancement, and microabscesses were observed in the basal ganglia, subcortical white matter, and left anterior thalamic area. On T2-weighted images, the large hypointense lesion caused a minimal mass effect, which was accompanied by significant peripheral edema (Figure 1). Diffusion-weighted imaging revealed several diffusely localized lesions with restricted diffusion in both hemispheres and the cerebellum (Figure 2). Multivoxel MR spectroscopy revealed significant elevation in lipid and lactate levels, indicating an abscess. A brain biopsy was performed and pathological examination confirmed the diagnosis of Aspergillus infection (Figure 3, arrowheads). The patient was treated with intravenous liposomal amphotericin B but died despite a 57-day course of antifungal therapy. Intracranial Aspergillus infection is a rare condition that is difficult to diagnose because of the lack of specific imaging features^1^ ^-^ ^3^.
